# A Dictionary of Dental Science and Such Words and Phrases of the Collateral Sciences as Pertain to Art and Practice of Dentistry

**Published:** 1899-06

**Authors:** 


### Bibliographical.

---

### A DICTIONARY OF DENTAL SCIENCE AND SUCH WORDS AND PHRASES OF THE COLLATERAL SCIENCES AS PERTAIN TO ART AND PRACTICE OF DENTISTRY.

---

CHAPIN A. HARRIS, M. D., D. D. S.

---

Sixth Edition, Carefully Revised and Enlarged by FERDINAND J. S. GORGAS, M. D., D. D. S., Author of "Dental Medicine;" Editor of Harris' "Principles and Practice of Dentistry;" Professor of Principles of Dental Science, Oral Surgery and Prosthetic Dentistry in the University of Maryland.

---

PHILADELHHIA, P. BLAKISTON, SON & CO., 1898.

---

Nothing is more important to both student and practitioner than a reliable, scientific, up-to-date dictionary. The different editions of Harris' dictionary of Dental Science are part of the history of the profession.

In the sixth edition, Prof. Gorgas has given to us the result of the last seven years of work. The book is greatly enlarged by the edition of thousands of words for which purpose he has delved freely into the allied sciences wherever their services are brought into use by the dentist. The only criticism that can be made of this work is that Prof Gorgas has not kept within the bounds of a dictionary, simply a description of words and phrases, but strays freely into the paths that belong to works on practice and therapeutics in describing treatment and remedies. In the condensed form in which this must be done in a work of this chrracter, it may easily be productive of more harm than good.—*Items of Interest.*

---

